# Poster Session I - A114 OUT OF SCOPE: EVALUATING THE QUALITY, READABILITY, AND AGE-INCLUSIVITY OF ONLINE COLONOSCOPY EDUCATION FOR YOUNGER ADULTS

**DOI:** 10.1093/jcag/gwaf042.114

**Published:** 2026-02-13

**Authors:** A S Hassan, A Datta, A Zoughlami, A Saed Aldien, T Bessissow, G Wild

**Affiliations:** McGill University Faculty of Medicine and Health Sciences, Montreal, QC, Canada; McGill University Faculty of Medicine and Health Sciences, Montreal, QC, Canada; Division of Gastroenterology and Hepatology, McGill University Health Centre, Montreal, QC, Canada; McGill University Faculty of Medicine and Health Sciences, Montreal, QC, Canada; Division of Gastroenterology and Hepatology, McGill University Health Centre, Montreal, QC, Canada; Division of Gastroenterology and Hepatology, McGill University Health Centre, Montreal, QC, Canada

## Abstract

**Background:**

The incidence of colorectal cancer (CRC) is increasing among adults under age 50, yet most online educational materials about colonoscopy remain targeted at older populations. Effective resources must be high-quality, readable, and age-relevant to support informed screening decisions among younger adults.

**Aims:**

To assess the quality, readability, comprehensiveness, and age-inclusivity of online colonoscopy education materials targeting younger adults.

**Methods:**

Google searches were conducted in private browsing mode using three predefined queries. The first 30 English-language results per term were screened, yielding 54 unique websites. Inclusion criteria required patient-directed colonoscopy education content. Two reviewers assessed quality using the DISCERN instrument and JAMA Benchmark criteria. Readability was calculated using five validated tools. Adapted from the American Medical Association (AMA) informed consent guidelines, comprehensiveness was defined by the inclusion of the procedure’s purpose, risks, and alternatives. Age inclusivity was defined as any reference to adults under age 50.

**Results:**

The mean DISCERN score was 60.5 ± 12.2, indicating good overall quality across websites. The mean JAMA score was 1.44 ± 1.0. The average reading level was grade 10, above AMA-recommended sixth-grade level, and the mean Flesch Reading Ease score was 53.1, indicating “fairly difficult” readability (score range: 50 - 59). Only six websites (11.1%) included all three informed consent elements, and fewer than half (42.6%) referenced adults under 50. Quality and readability metrics were independent of one another.

**Conclusions:**

Online colonoscopy resources show high content quality but are limited by moderate comprehensiveness, high reading grade levels, and poor age inclusivity. Improved readability and targeted messaging are essential to support informed screening decisions among younger adults.

**Funding Agencies:**

None

